# Oral Anticoagulant Adequacy in Non-Valvular Atrial Fibrillation in Primary Care: A Cross-Sectional Study Using Real-World Data (Fantas-TIC Study)

**DOI:** 10.3390/ijerph18052244

**Published:** 2021-02-24

**Authors:** M. Rosa Dalmau Llorca, Carina Aguilar Martín, Noèlia Carrasco-Querol, Zojaina Hernández Rojas, Emma Forcadell Drago, Dolores Rodríguez Cumplido, Josep M. Pepió Vilaubí, Elisabet Castro Blanco, Alessandra Q. Gonçalves, José Fernández-Sáez

**Affiliations:** 1 Equip d’Atenció Primària Terres de l’Ebre, Institut Català de la Salut, Tortosa, 43500 Tarragona, Spain; rdalmau.ebre.ics@gencat.cat (M.R.D.L.); zojahernandez@gmail.com (Z.H.R.); eforcadellg.ebre.ics@gencat.cat (E.F.D.); jmpepio.ebre.ics@gencat.cat (J.M.P.V.); 2 Grupo GAVINA, Campus Terres de l’Ebre, Universitat Rovira i Virgili, Tortosa, 43500 Tarragona, Spain; elicasblan@gmail.com; 3 GAVINA Research Group, Tortosa, 43500 Tarragona, Spain; caguilar.ebre.ics@gencat.cat (C.A.M.); aqueiroga.ebre.ics@gencat.cat (A.Q.G.); jfernandez@idiapjgol.info (J.F.-S.); drfif@gmail.com (D.R.C.); 4 Unitat de Suport a la Recerca Terres de l’Ebre, Fundació Institut Universitari per a la Recerca a l’Atenció Primària de Salut Jordi Gol i Gurina (IDIAPJGol), Tortosa, 43500 Tarragona, Spain; 5 Unitat d’Avaluació, Direcció d’Atenció Primària Terres de l’Ebre, Institut Català de la Salut, Tortosa, 43500 Tarragona, Spain; 6 Hospital Universitari de Bellvitge, Institut Català de la Salut, 08907 Barcelona, Spain; 7 Unitat Docent de Medicina de Familia i Comunitària, Tortosa-Terres de l’Ebre, Institut Català de la Salut, Tortosa, 43500 Tarragona, Spain

**Keywords:** atrial fibrillation, direct oral anticoagulants, renal function, time in therapeutic range, vitamin K antagonists

## Abstract

**Background**: Oral anticoagulants (OAs) are the treatment to prevent stroke in atrial fibrillation (AF). Anticoagulant treatment choice in non-valvular atrial fibrillation (NVAF) must be individualized, taking current guidelines into account. Adequacy of anticoagulant therapy under the current criteria for NVAF in real-world primary care is presented. **Methods**: Cross-sectional study, with real-world data from patients treated in primary care (PC). Data were obtained from the System for the Improvement of Research in Primary Care (SIDIAP) database, covering 60,978 NVAF-anticoagulated patients from 287 PC centers in 2018. **Results**: In total, 41,430 (68%) were treated with vitamin K antagonists (VKAs) and 19,548 (32%) NVAF with direct-acting oral anticoagulants (DOACs). Inadequate prescription was estimated to be 36.0% and 67.6%, respectively. Most DOAC inadequacy (77.3%) was due to it being prescribed as a first-line anticoagulant when there was no history of thromboembolic events or intracranial hemorrhage (ICH). A total of 22.1% had missing estimated glomerular filtration rate (eGFR) values. Common causes of inadequate VKA prescription were poor control of time in therapeutic range (TTR) (98.8%) and ICH (2.2%). **Conclusions**: Poor adequacy to current criteria was observed, being inadequacy higher in DOACs than in VKAs. TTR and GFR should be routinely calculated in electronic health records (EHR) to facilitate decision-making and patient safety.

## 1. Introduction

Oral anticoagulants (OAs) are used to prevent stroke in atrial fibrillation (AF). The American guidelines recommend using warfarin, a vitamin K antagonist (VKA), and direct-acting oral anticoagulants (DOACs) to prevent stroke in AF in patients with CHA2DS2-VASC ≥ 2, taking into account individual risk/benefit of bleeding [1]. The European Society of Cardiology guidelines recommend the use of DOACs as first-line treatment for non-valvular atrial fibrillation (NVAF) [2].

In Catalonia, anticoagulant therapy for NVAF follows the “Informe de Posicionamiento terapéutico” (IPT) (Therapeutic Positioning Report) and the “Pautes per a l’harmonització del tractament” (PHT) (Therapeutic Harmonization Guidelines for the Use of Oral Anticoagulants) [3,4] from the Ministry of health, social services and equality of Spain and the Catalan Health Service, respectively. Under these guidelines, VKA are the first-line treatment for AF cases requiring anticoagulation, while DOAC are used in specific situations of NVAF [4].

The various DOACs are at least as effective as warfarin at preventing stroke in NVAF. The four DOACs (dabigatran, rivaroxaban, apixaban, and edoxaban) commercialized up to 2017 in Spain, and currently in many European countries, reduce the rates of stroke, systemic embolism, major bleeding, intracranial hemorrhage (ICH), cardiovascular mortality and total mortality, but are associated with a higher risk of intestinal bleeding [5,6,7,8]. Despite the dosage and interaction advantages of DOACs over VKAs, physicians individually evaluate the choice of anticoagulant [3,4]. Since VKAs remain the first-line treatment for NVAF in Spain, prescription of DOACs in Spain is amongst the lowest in Europe [9], although DOAC prescription is currently increasing. However, there is a trend towards inadequate OA prescription in NVAF patients that was not yet sufficiently described with population data [10]. 

This study analyzes the adequacy of the anticoagulant therapy prescription (VKAs and DOACs) in NVAF under the current recommendation criteria in primary care (PC) Catalan population, using real-world health care data. 

## 2. Material and Methods

### 2.1. Study Design

A cross-sectional study with real-world data of primary care patients of the Catalan Institute of Health (ICS) was conducted. The study included patients with NVAF diagnoses in 2018 at the 287 ICS primary care centers (PCCs). These PCCs are responsible for the care of an estimated 5,564,292 people (80% of the Catalan and >10% of the Spanish populations), and employ 3384 physicians. 

### 2.2. Data Source

**Figure 1:**
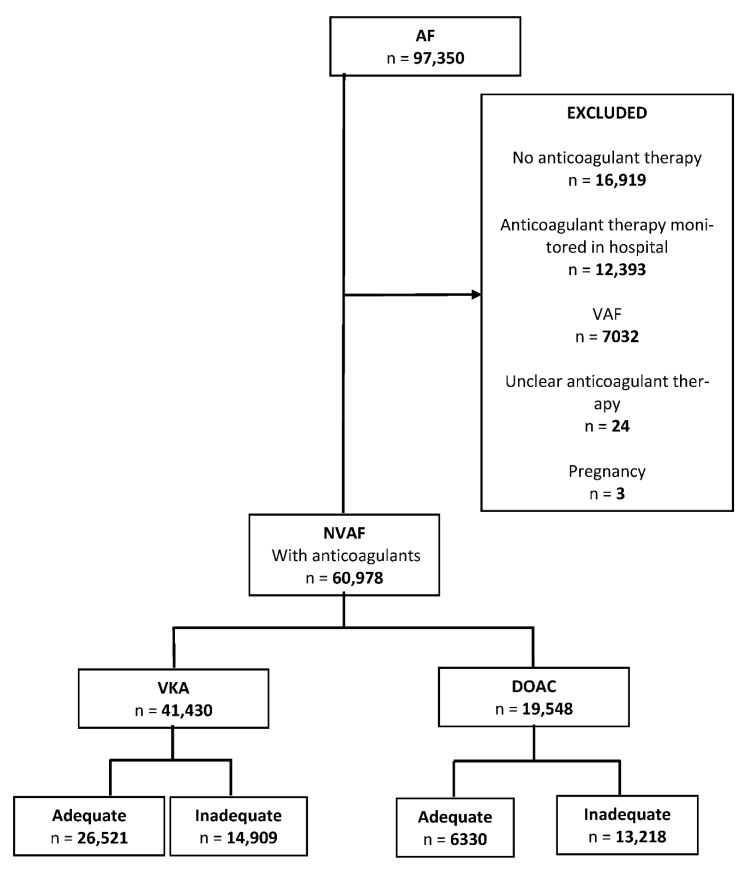
Flowchart of the study.

Data were obtained from the SIDIAP (Information System for Research in Primary Care) population database, which is representative of the Catalan population [11,12]. We identified 97,350 patients in the SIDIAP with a diagnosis of AF for at least 12 months (Figure 1). Patients with an active prescription for an anticoagulant on 1 January 2018 were included. We considered all authorized anticoagulant treatments with VKAs (acenocoumarol and warfarin) and DOACs (dabigatran, rivaroxaban, apixaban, and edoxaban) in Spain in 2016. Drug data based on Anatomic Therapeutic Chemical (ATC) codes were collected [13].

SIDIAP contains anonymized clinical information from various data sources [11,12]: (1) electronic health records (EHRs) from ICS primary care (known as eCAP—*Estació Clínica d’Atenció Primària*), which, since 2006, has included information on sociodemographic characteristics, health conditions registered as International Classification of Diseases (ICD) 10 codes [14], general practitioner prescriptions and clinical parameters; (2) laboratory data; (3) prescription data, available since 2005, with information on all pharmaceutical products dispensed by community pharmacies of the Catalan Health System, based on ATC classification system codes [13].

## 3. Study Population

### Inclusion and Exclusion Criteria

We included patients who received OA treatment and monitored the anticoagulant therapy in PCCs of the ICS, diagnosed with NVAF one year before the study date, and with at least six controls of the International Normalized Ratio (INR) during the year before the study. This restriction minimizes INR variability at the start of the treatment and avoids the effect of temporary withdrawal of VKAs in patients with good control of INR. 

Patients were considered to have been exposed to anticoagulation if they were prescribed anticoagulants (acenocoumarol, warfarin, dabigatran, rivaroxaban, apixaban, or edoxaban) for at least 2 months before the study date. The anticoagulant medication included in the study was those started the closest to the study date. 

We excluded patients with no OA therapy, patients whose OA therapy was monitored in hospital, those with valvular atrial fibrillation (mitral stenosis or with a mechanical prosthetic valve), pregnant women, and patients whose anticoagulant treatment at the beginning of the study could not be ascertained (Figure 1).

## 4. Study Variables

*Main variable.* Adequacy of anticoagulant treatment (VKAs and DOACs) under IPT criteria (Table 1).

*Adequacy of VKA prescription.* Patients receiving a VKA who met one of the following conditions [4]: TTR^R^ ≥ 65% (according to Rosendaal’s formula [15]), TTR^R^ < 65% and glomerular filtration (_e_GFR estimated using CKD-EPI) < 15/min/1.73 m^2^, history of adverse drug reaction (ADR) to DOACs (including allergy), a missing value of _e_GFR or TTR^R^. Inadequate prescription of VKAs was concluded for patients receiving a VKA who met one of the following conditions: history of ADR to VKAs or intracranial hemorrhage (ICH) with _e_GFR > 15/min/1.73 m^2^. 

*Adequacy of DOAC prescription.* Patients who received one DOAC and met one of the following conditions [4]: pre-DOAC TTR^R^ < 65% if the first OA was a VKA, or ADR to VKA, thromboembolic event (TEE) after starting VKA with TTR ≥ 65% (post-VKA TTE), ICH or stroke with a high risk of bleeding (SHRB). Inadequate prescription of DOACs was concluded for patients receiving DOACs who met one of the following conditions: ADR to DOAC, _e_GFR < 15/min/1.73 m^2^, or a missing _e_GFR value, or when the DOAC was prescribed as first-line anticoagulant with no history of post-VKA TTE, SHRB or ICH. 

*Secondary variables.* Sociodemographic variables, type of anticoagulant treatment, place of OA prescription, history of cardiovascular disease (CVD), ICH, morbidity, gastrointestinal hemorrhage (GH), history of high risk of bleeding (HRB) and other hemorrhages, estimated scores [15,16,17,18] based on participants’ real world data (Table 2) and calculated constructed variables (see Table 3). Diseases were classified as specified in the ICD10 code list [14].

## 5. Statistical Analysis

Anonymized data were exported from the SIDIAP database to the Statistical Package of Social Sciences IBM (SPSS) version 20.0. As this was a population-based study, the sample guaranteed ≥99% statistical power. Data were cleaned, taking into account the minimum and maximum values of variables, and an analysis of missing values was carried out. Variables were summarized as the mean of frequencies and percentages for categorical variables, and the median and interquartile range for continuous variables. Variables were created to describe adequacy according to IPT criteria [4] (Table 1). Two-proportion Z-tests were conducted to detect significant differences between proportions of categories of the variables describing inadequate prescription of VKAs and DOACs; significance was concluded for values of *p* < 0.05.

## 6. Results

The SIDIAP database contained 97,350 adult patients with active AF identified in 2018 (Figure 1). Of these, 60,978 were patients with NVAF who were receiving anticoagulant therapy and who fulfilled the inclusion criteria. The mean age was 78 years (SD = 9 years) and 50.7% were men. The sociodemographic characteristics, cardiovascular risk factors and morbidities of the study patients, including percentages with missing data, are summarized in Table 4. 

The main study variable is OA adequacy. OA adequacy characteristics, based on the official adequacy criteria [3,4], are described.

Regarding OA therapy, 41,430 (68%) had VKA prescription and 19,548 (32%) had DOAC prescription (Table 5).

The main outcome variable was OA adequacy, based on the following official adequacy criteria [3,4]:

### 6.1. Adequacy and Inadequacy to DOAC Prescription

Inadequacy of DOAC prescription was 67.6%, versus 32.4% of adequacy (Table 5). Inadequate prescription was significantly higher in men (68.4%) than in women (66.8%). Up to 82% of people <60 years of age who were treated with DOACs did not meet prescription criteria. Moreover, inadequate DOAC prescription inversely associated with age (Table 5). Up to 69–76.4% patients with no history of CVD, ICH, morbidity or with an established history of HRB failed to meet the adequacy criteria for DOACs. Inadequate prescription increased inversely with the CHA_2_DS_2_VASc and HAS-BLED scores, a value of zero being that most commonly associated with inadequate prescription (Table 5).

Most patients (77.3%) had a _e_GFR ≥15, although this did not fulfill the adequacy criteria for other reasons, for example, because the DOAC was prescribed as the first-line anticoagulant without histories of post-VKA TEE, SHRB or ICH, or of ADR to DOAC (Table 6). This treatment is inadequate for patients with _e_GFR < 15 or with a missing GF value (22.1%) (Table 6).

DOAC prescription was 32.4% adequate (Table 6). Adequate prescription was concluded for all patients presenting _e_GFR ≥ 15 and 58.4% patients with a pre-DOAC TTR^R^ of <65% (who were prescribed a VKA as first-line treatment). In total, 40.9%, 27.7% and 6.4% of the adequate patients had post-VKA TEE, SHRB and ICH, respectively (see detail in Supplementary Material).

### 6.2. Adequacy and Inadequacy to VKA

The adequacy and inadequacy of the VKA prescription were 64% and 36%, respectively (Table 5). Inadequate prescription was significantly higher in women (37%) than in men (34.2%) (Table 5). Adequacy criteria for VKA therapy were not met in 36.2–41.8% patients treated with VKAs with a history of CVD (except for aortic atherosclerosis), any of the studied morbidities or an established history of HRB. Inadequate prescription was also associated with ICH, and 90.4% patients with ICH were inadequately prescribed this treatment (Table 5). Inadequate prescription increased with HAS-BLED scores, reaching 64.4% when HAS-BLED was ≥4. Patients with CHA_2_DS_2_VASc ≥ 4 had significantly higher rates of inadequacy than those with lower CHA_2_DS_2_VASc values. Institutionalized patients and those in domiciliary care had significantly higher inadequacy rates (43.3–44.9%) than did patients who were able to attend the PPC.

Most of the VKA inadequacy (98.8% of cases) was related to VKA prescription when TTR^R^ was <65% after 6 months of treatment (Table 7).

Adequacy criteria for VKAs were met in 64% of cases (Table 7). Patients with TTR^R^ < 65% were adequately prescribed a VKA because of a missing _e_GFR value, _e_GFR < 15 or ADR to DOACs. Most were adequately prescribed with respect to TTR^R^ ≥ 65% (94.8%), but 170 had ICH and seven presented ADR to VKAs. 

## 7. Discussion

This population-based study demonstrates the adequacy of anticoagulant treatment according to IPT [4] criteria in PC patients with NVAF in 2018 in Catalonia. To our knowledge, this is the first real-word data study with a large cohort of Catalan population that evaluates prescription adequacy of anticoagulant therapy for patients with NVAF in relation to IPT [4] criteria (also incorporated into the PHT [3]). Inadequate treatment was more common for DOACs (67.6%) than for VKAs (36%). Most of the inadequacy of DOAC prescription arose because it was prescribed as first-line anticoagulant when there was no history of TEEs or ICH. Another substantial part of the inadequacy stems from missing _e_GFR values. Common causes of inadequate VKA prescription were poor TTR control and some cases of ICH. 

The European Society of Cardiology [2] and the American Heart Association [1] were prompted to recommend the use of DOACs as first-line treatment instead of VKAs, because the results of pivotal clinical trials with DOACs [5,6,7,8,19] showed a reduction in the incidence of stroke by at least the same degree as produced by warfarin, and presented a lower rate of ICH. 

More recently, some real-world effectiveness studies have highlighted the limitations of the pivotal DOAC clinical trials, showing that DOACs and warfarin have similar effectiveness and safety with respect to TTE and severe non-intracranial hemorrhages [20,21]. Some studies also showed similar bleeding ratios for DOACs and VKAs [22]. Conversely, other studies with real-world data that compared warfarin and DOACs underlined the effectiveness of DOACs for preventing TTE and reducing the risk of intracranial hemorrhage [23,24]. To resolve these conflicting results, health institutions require cost-effectiveness studies based on real-world data that analyze DOACs and VKAs in their specific settings. Recent studies have shown that DOACs are more cost-effective for patients who are poorly controlled with VKAs and for patients at high risk of thromboembolism and bleeding [25,26], supporting the IPT’s recommendations concerning DOAC use in specific situations.

In our study, control was poor (TTR < 65%) in 38.8% patients who received VKAs, similar to the levels in other regions of Spain (39.4%) [27]. TTR was calculated based on the INR and the timings registered in the EHRs. Poor control of anticoagulation is associated with increased risks of stroke, bleeding and all-cause mortality [28,29]. Therefore, good control of the INR is essential for the patient, but often not achieved in Catalonia [27], Spain or other countries [30]. Typically, patients treated with VKAs are infracoagulated and not so much overcoagulated, thus increasing the risk of TTE and not so much the bleeding risk. Management of VKAs outside of clinical trials is associated with poor control, particularly at the start of VKA therapy. Good access to TTR values in PC would improve the control of patients treated with VKAs and decision-making regarding adequate switching to DOACs [31], although most PCCs in Spain do not currently have access to such information. On the other hand, patients who receive VKAs and have had HIC should be considered for switching to DOAC as this has been shown to be superior in preventing ICH.

Measurement of GFR is essential for choosing anticoagulant therapy. The absence of this information leads to inadequate prescribing of DOAC. In clinical practice, various equations estimate the GFR [32], one of the most commonly used for DOACs being the CKD-EPI [18]. This was developed to improve the estimate, and is the one we use in our own setting. Not all PCCs include this calculated formula in their EHRs, which hinders the determination of the correct DOAC dose and the transition between VKAs and DOACs. Importantly, while VKAs can be used regardless of the GFR value, it can determine the adequacy in the case of DOACs [4]. For instance, they are contraindicated in some cases. Dabigatran cannot be used with _e_GFR <30 mL/min, and rivaroxaban, apixaban and edoxaban when _e_GFR is <15 [4]. In this study it was not differentiated the DOAC type and therefore eGFR <15 was considered to be the contraindication criterion of most DOAC. PCCs can access hospital creatinine results, and the number of laboratories providing GFR calculations to adjust anticoagulants is increasing. In this study, we used creatinine to estimate GFR. Records of a substantial proportion of patients receiving DOACs lack a measurement of creatinine from the previous year. It was not possible to estimate their _e_GFR, which is essential for deciding the prescription and for adjusting the DOAC treatment dose. Probably, the lack of experience on DOACs management, in our setting, explains the absence of _e_GFR in patients receiving this treatment. This study wants to provide evidence for this problem. While VKA treatments can be adjusted without recourse to renal function information, this is not possible for DOACs. The increase and decrease of DOAC plasma levels, which are closely related to renal function, are associated with hemorrhagic and ischemic events, respectively [33]. Thus, _e_GFR is essential to initiate dosing and to monitor the appropriate dose of DOAC, avoiding problems with DOACs underdoses and overdoses. DOAC dose adjustment is based primarily on _e_GFR, and other factors such as age, weight and interactions with other drugs. 

A report from our setting concerning the use of anticoagulants in NVAF, based on 2014–2017 data, showed the same usage of VKAs (69%) and DOACs (31%) as in our current study (VKAs, 68%; DOACs, 32%) [34]. The same study also showed that 29.5% of patients treated with DOACs lacked clinical data with which to evaluate the dosage correctly [34]. Measures are currently being evaluated and used to increase the availability of GFR determinations in PCCs of the ICS for patients treated with DOACs. To ensure that dosage determination takes kidney function into account, each physician can check their patients who are treated with a DOAC, with no measurement of _e_GFR recorded during the previous 12 months.

A history of ICH should be considered when prescribing DOACs [4], and treatment should be personalized. Clinical trials have shown the effectiveness of DOACs compared with warfarin at reducing stroke and mortality. DOACs involve a similar risk of major bleeding as with warfarin, but a lower risk of ICH and a higher risk of intestinal bleeding [35].

Results of clinical trials are often difficult to translate into clinical practice, since patients often have more comorbidities, take more medication and cannot be followed up so closely. With regard to ICH, effectiveness studies with real-world data have confirmed that DOACs have fewer associated risks than VKAs [23], so the IPT advises DOAC treatment in such cases [4]. Most patients with a history of ICH received DOACs, probably because they were started on them after this event.

Studies also reveal the risk of gastrointestinal hemorrhage with DOACs [5,19]. A history of gastrointestinal hemorrhage is more common in patients treated with DOACs than with VKAs. Even though gastrointestinal hemorrhage is not a criterion in the IPT, clinicians need to consider this risk and advise their patients accordingly.

This study provides a real-world snapshot of anticoagulation in patients with NVAF. We consider that these recently acquired data on adequacy with respect to the criteria in force in Catalonia and Spain could be generalized to other geographical areas. Our results highlight the urgency of facilitating access to TTR by all specialists who manage anticoagulants, and the need for renal function measurements when DOACs are prescribed. It also shows that DOACs are frequently prescribed to young patients who have not previously experienced either the TEE or ICH that would justify this choice. 

The study has some limitations. First, the cross-sectional design, used to investigate current patterns, does not allow causal inferences to be made. Nevertheless, it serves to generate hypotheses that could be examined in further studies. Second, there was a high frequency of missing scores, which had to be calculated from available patient data. Type of reporting and underreporting might constitute a limitation in this real-word data based study. The IPT does not take into account treatment adherence or lack of access to INR monitoring. However, access to INR is very high in PC patients of the ICS, considering that patients who are unable to attend the PCC are monitored at home. Guidelines agreed by experts and based on scientific evidence are key instruments for making individual decisions, although other factors, such as compliance and adherence to drugs, must frequently be considered. Both, compliance with VKA and adherence to DOAC are key factors in oral anticoagulant treatment in NVAF and should be consider in further studies.

The Spanish IPT criteria are more restrictive with respect to the use of DOACs than the European guidelines, although both are supported by scientific evidence. Regarding the main factors associated with inadequate OA prescription, our study highlights the need for improved strategies that can be generalized to other locations. The OA prescription criteria of different countries’ guidelines can differ in aspects related to first-line treatment or specific situations when the use of DOACs or VKAs is recommended. However, in clinical practice, the adherence to official criteria guidelines improves patient safety.

## 8. Conclusions

In patients with NVAF, prescription adequacy was higher for VKAs than for DOACs. Most of the inadequacy of DOACs arises from its prescription as first-line anticoagulant in patients without a history of TEEs or of ICH. A substantial proportion of the inadequacy is also explained by missing _e_GFR values. Common causes of inadequate VKA prescription were poor TTR control and some cases of ICH.

TTR and _e_GFR should be routinely calculated and recorded in the EHRs to facilitate decision-making. Main factors associated with inadequate OA prescription and the improved strategies identified in the present study are useful for improving clinical practice and patient safety in the health systems of different countries.

## Figures and Tables

**Table 1:** Catalan (PTH) and IPT Spanish (IPT) criteria for oral anticoagulant therapy.

VKAs (acenocoumarol and warfarin) are the first-line therapy in patients newly diagnosed with NVAF (except for patients meeting criteria for DOACs)
DOACs are the first-line therapy in patients with NVAF in the following situations:
Patients with known hypersensitivity or specific contraindication to VKAs.
History of intracranial hemorrhage PHT (if benefits outweigh risks).
Ischemic stroke with clinical and neuroimaging criteria of high risk of ICH (at least one of: widespread leukoaraiosis and/or multiple cortical microbleeds; and HAS-BLED ≥ 3) for whom benefits of starting anticoagulation outweigh the risks of hemorrhage.
Patients receiving a VKA with thromboembolic events even with INR values within the therapeutic range.
Patients receiving a VKA with poor INR control (range 2–3) despite good adherence. Good control is TTR ≥65% calculated by the Rosendaal method.
No access to INR control.

**Abbreviations:** IPT: Therapeutic Positioning. Report UT_DAOA/V5/211122016; PHT: Guidelines for the Therapeutic Harmonization in the use of oral anticoagulants with Atrial Fibrillation; VKA: vitamin K antagonist; DOACs: direct oral anticoagulants; **NVAF**: non-valvular atrial fibrillation; INR: International Normalized Ratio; TTR: time in therapeutic range; ICH: intracranial hemorrhage; HAS-BLED: bleeding risk score.

**Table 2:** Scores evaluated in the present study.

Score	Definition	Reference
CHA_2_DS_2_-VASC	Score based on having or not having diagnosed heart failure (HF) and/or Teichholz ejection fraction <40%, HT, DM, stroke, IC or PA, or aortic atherosclerosis, age between 65 and 74 or >75 years. Annual scores from 2011 to 2017 were obtained.	[16]
HAS-BLED	Score based on having or not having systolic blood pressure >160 mmHg, kidney failure or CKD-EPI <45 in two consecutive measurements, LF, stroke or ICH, HRB, Rosendaal TTR < 65%. Annual scores from 2011 to 2017 were obtained.	[17]
TTR	Calculated by the Rosendaal method, which yields the percentage of the time the patient is within the 2–3 range, assuming a linear progression between the consecutive INR values and calculating the daily specific INR. Poor control is concluded when TTR <65%. Two annual values of 12 and 6 months (last months of the year) from 2011 to 2017 were obtained.	[15]
CKD-EPI(mL/min/1.73 m^2^)	_e_GFR is based on creatinine and the Chronic Kidney Disease Epidemiology Collaboration equation (CKD-EPI). Annual values from 2011 to 2017 were obtained if there was a creatinine value that year, otherwise it was considered a missing value. If there was more than one creatinine value per year, the lowest value was considered.	[18]

HT: hypertension; DM: diabetes mellitus; IC: ischemic cardiomyopathy; PA: peripheral arteriopathy; LF: liver failure; HRB: high risk of bleeding; INR: International Normalized Ratio; TTR: time in therapeutic range according to the Rosendaal method; _e_GFR: glomerular filtration according to CKD-EPI (mL/min/1.73 m^2^) score; ICH: intracranial hemorrhage.

**Table 3:** Definition of the variables evaluated in the present study.

Variable	Definition
**Thromboembolic event (TTE)**	Includes IC, PA, aortic atherosclerosis, ischemic stroke, including TIA
**Thromboembolic event after starting VKA with TTR ≥ 65% (post-VKA TTE)**	Includes IC, PA, aortic atherosclerosis, ischemic stroke, including TIA, after starting a VKA
**Stroke with high risk of bleeding (SHRB)**	Includes ischemic stroke, including TIA in patients with HASBLED ≥ 3 or HRB
**TTR of 6 or 12 months (TTR)**	We calculated TTR for the 12 months before the cut-off date. If there was no measurement, we obtained that for 6 months
**_e_GFR**	Glomerular filtration in mL/min/1.73 m^2^, according to the Chronic Kidney Disease Epidemiology Collaboration equation (CKD-EPI) [18] for the previous 12 months, or for the lowest creatinine measurement during the previous 24 months. Categorized as ≥15 mL/min/1.73 m^2^ or <15 mL/min/1.73 m^2^
**Adverse drug reaction (ADR)**	For VKAs and DOACs, includes moderate and severe adverse reactions and allergies to the medication group, without specifying the active ingredient
**TTR before starting DOAC (pre-DOAC TTR)**	We calculated TTR before starting DOAC administration in patients who had taken VKAs and categorized values as ≥ 65% or < 65%
**First oral anticoagulant (First OA)**	The first OA prescribed was determined in patients who had taken VKAs or DOACs
**No event**	No post-VKA TTE, no ICH, no SHB

VKA: vitamin K antagonist; DOACs: direct oral anticoagulants; IC: ischemic cardiomyopathy; PA: peripheral arteriopathy; TIA: transient ischemic attack; HRB: high risk of bleeding; TTR: time in therapeutic range; **_e_GFR**: estimated glomerular filtration; ICH: intracranial hemorrhage.

**Table 4:** Sociodemographic study population characteristics, cardiovascular risks factors and comorbidities.

	Total	%
Total	60,978	
**Gender**		
Female	**30,085**	49.3
Male	**30,893**	50.7
**Age (years, mean, SD)**	**78 (9)**	
**Age (year range)**		
<60	**1966**	3.2
60–69	**6158**	10.1
70–79	**18,794**	30.8
≥80	**34,060**	55.9
**First OA prescribed**		
VKA	**52,914**	86.8
DOAC	**7974**	13.1
**OA prescription in PC**	**42,272**	69.3
**Cardiovascular history**		
Peripheral arteriopathy	**4193**	6.9
Ischemic cardiopathy	**11,622**	19.1
Aortic atherosclerosis	**634**	1.0
Ischemic stroke or TIA	**11,659**	19.1
**Intracranial hemorrhage**	**826**	1.4
**Morbidity**		
Diabetes mellitus	**20,061**	32.9
Arterial hypertension	**48,547**	79.6
Heart failure	**16,099**	26.4
Kidney failure	**17,621**	28.9
**History of bleeding risk**		
Alcohol	**2570**	4.2
Intracranial aneurysm	**63**	0.1
Portal hypertension	**104**	0.2
Liver failure	**381**	0.6
Hereditary telangiectasia	**4**	0.0
Active aneurysm and dissection of aorta	**936**	1.5
Gastrointestinal angiodysplasia	**177**	0.3
Hemorrhages other than digestive and intracranial	**838**	1.4
**Gastrointestinal hemorrhage**	**5138**	8.4
**Scores**		
CHA_2_DS_2_VASc		
0	**903**	1.5
1	**3720**	6.1
2	**10,723**	17.6
3	**20,501**	33.6
≥4	**25,131**	41.2
HAS-BLED		
0	**2016**	3.3
1	**22,145**	36.3
2	**21,640**	35.5
3	**10,947**	18.0
≥4	**4230**	6.9
_e_GFR mL/min/1.73 m^2^		
<15	**384**	0.6
15–29	**2839**	4.7
30–49	**12,212**	20.0
≥50	**39,447**	64.7
Missing	**6096**	10.0
**Patients visited outside PPC**		
Domiciliary care	**7832**	12.8
Institutionalized	**2662**	4.4

VKA: vitamin K antagonist; DOAC: direct oral anticoagulant; **OA**: oral anticoagulant; **PC**: primary care; TIA: transient ischemic attack; **PPC**: primary care centre; _e_GFR: glomerular filtration estimated by CKD-EPI (mL/min/1.73 m^2^).

**Table 5:** Inadequate prescribing of VKAs and DOACs by patient characteristics.

	Inadequate Prescribing of VKAs				Inadequate Prescribing of DOACs			
	Total	n	%	^a^ p	Total	n	%	^a^ p
**Total**	**41,430**	14,909	36.0		**19,548**	13,218	67.6	
**Gender**								
Female	**20,285**	7671	37.8	<0.001	**9800**	6550	66.8	0.019
Male	**21,145**	7238	34.2		**9748**	6668	68.4	
**Age**								
<60 ^b^	**935**	347	37.1		**1031**	851	82.5	
60–69	**3774**	1303	34.5	0.138	**2384**	1808	75.8	<0.001
70–79	**12,893**	4400	34.1	0.063	**5901**	4090	69.3	<0.001
≥80	**23,828**	8859	37.2	0.967	**10,232**	6469	63.2	<0.001
**First OA prescribed**								
VKA	**40,671**	14,568	35.8	0.385	**12,243**	6425	52.5	<0.001
DOAC	**669**	251	37.5		**7305**	6793	93.0	
**OA prescription in PC**								
Yes	**33,263**	11,861	35.7	0.005	**9009**	6568	72.9	<0.001
No	**8167**	3048	37.3		**10,539**	6650	63.1	
**Cardiovascular history**								
Peripheral arteriopathy	**2704**	1090	40.3	<0.001	**1489**	710	47.7	<0.001
No	**38,726**	13,819	35.7		**18,059**	12,508	69.3	
Ischemic cardiomyopathy	**7514**	2876	38.3	<0.001	**4108**	2269	55.2	<0.001
No	**33,916**	12,033	35.5		**15,440**	10,949	70.9	
Aortic atherosclerosis	**400**	144	36.0	0.995	**234**	119	50.9	<0.001
No	**41,030**	14,765	36.0		**19,314**	13,099	67.8	
Ischemic stroke or TIA	**6768**	2576	38.1	<0.001	**4891**	2025	41.4	<0.001
No	**34,662**	12,333	35.6		**14,657**	11,193	76.4	
**Intracranial hemorrhage**	**364**	329	90.4	<0.001	**462**	58	12.6	<0.001
No	**41,066**	14,580	35.5		**19,086**	13,160	69.0	
**Morbidity**								
Diabetes mellitus	**13,587**	5412	39.8	<0.001	**6474**	3939	60.8	<0.001
No	**27,843**	9497	34.1		**13,074**	9279	71.0	
Arterial hypertension	**33,360**	12,085	36.2	0.038	**15,187**	9934	65.4	<0.001
No	**8070**	2824	35.0		**4361**	3284	75.3	
Heart failure	**10,846**	4348	40.1	<0.001	**5253**	3232	61.5	<0.001
No	**30,584**	10,561	34.5		**14,295**	9986	69.9	
Kidney failure	**12,340**	4760	38.6	<0.001	**5281**	3117	59.0	<0.001
No	**29,090**	10,149	34.9		**14,267**	10,101	70.8	
**History of bleeding risk**								
Alcohol	**1727**	722	41.8	<0.001	**843**	518	61.4	<0.001
No	**39,703**	14,187	35.7		**18,705**	12,700	67.9	
Intracranial aneurysm	**28**	12	42.9	0.449	**35**	8	22.9	<0.001
No	**41,402**	14,897	36.0		**19,513**	13,210	67.7	
Portal hypertension	**80**	28	35.0	0.854	**24**	17	70.8	0.736
No	**41,350**	14,881	36.0		**19,524**	13,201	67.6	
Liver failure	**263**	111	42.2	0.035	**118**	80	67.8	0.967
No	**41,167**	14,798	35.9		**19,430**	13,138	67.6	
Hereditary telangiectasia	**2**	1	50.0	0.680	**2**	2	100.0	0.328
No	**41,428**	14,908	36.0		**19,546**	13,216	67.6	
Active aneurysm and dissection of aorta	**614**	219	35.7	0.869	**322**	202	62.7	0.059
No	**40,816**	14,690	36.0		**19,226**	13,016	67.7	
Gastrointestinal angiodysplasia	**92**	41	44.6	0.086	**85**	57	67.1	0.912
No	**41,338**	14,868	36.0		**19,463**	13,161	67.6	
Hemorrhages other than digestive and intracranial	**569**	202	35.5	0.808	**269**	165	61.3	0.027
No	**40,861**	14,707	36.0		**19,279**	13,053	67.7	
**Gastrointestinal hemorrhage**	**3286**	1280	39.0	<0.001	**1852**	1150	62.1	<0.001
No	**38,144**	13,629	35.7		**17,696**	12,068	68.2	
**Scores**								
CHA_2_DS_2_VASc								
0 ^b^	**404**	127	31.4		**499**	450	90.2	
1	**2107**	661	31.4	0.980	**1613**	1365	84.6	0.002
2	**7251**	2321	32.0	0.810	**3472**	2695	77.6	<0.001
3	**14,681**	5138	35.0	0.138	**5820**	4092	70.3	<0.001
≥4	**16,987**	6662	39.2	0.002	**8144**	4616	56.7	<0.001
HAS-BLED								
0 ^b^	**953**	156	16.4		**1063**	1003	94.4	
1	**14,563**	2343	16.1	0.819	**7582**	6478	85.4	<0.001
2	**15,112**	6158	40.7	<0.001	**6528**	4243	65.0	<0.001
3	**7783**	4308	55.4	<0.001	**3164**	1181	37.3	<0.001
≥4	**3019**	1944	64.4	<0.001	**1211**	313	25.8	<0.001
_e_GFR mL/min/1.73 m^2^								
<15 ^c^	**313**				**71**	71	100.0	
15–29 ^d^	**1850**	920	49.7		**989**	485	49.0	<0.001
30–49	**8239**	3616	43.9	<0.001	**3973**	2159	54.3	<0.001
≥50	**27,855**	10,373	37.2	<0.001	**11,592**	7580	65.4	<0.001
Missing	**3173**				**2923**	2923	100.0	
**Patients visited outside PPC**								
Domiciliary care	**4926**	2134	43.3	<0.001	**2906**	1696	58.4	<0.001
No	**36,504**	12,775	35.0		**16,642**	11,522	69.2	
Institutionalized	**1646**	739	44.9	<0.001	**1016**	602	59.3	<0.001
No	**39,784**	14,170	35.6		**18,532**	12,616	68.1	

**VKAs**: vitamin K antagonists; **DOACs**: direct oral anticoagulants; **OA**: oral anticoagulant; **PC**: primary care; TIA: transient ischemic attack; **PPC**: primary care centre; _e_GFR: glomerular filtration estimated by CKD-EPI (mL/min/1.73 m^2^). ^a^ Two proportion Z-test. ^b^ Reference in VKAs and DOACs. ^c^ Reference for DOACs only, ^d^ Reference for VKAs only.

**Table 6:** Adequate prescribing of direct oral anticoagulants (DOACs) based in the 2016 to the IPT criteria.

	Adequate Prescribing of DOACs		Inadequate Prescribing of DOACs	
	n	%	+n	%
Total	6330	32.4	13,218	67.6
_e_GFR				
<15			71	0.5
≥15	6330	100.0	10,224	77.3 ^a^
Missing			2923	22.1
**ADR DOAC**			55	0.4
**ADR VKA**	45	0.7	54	0.4
**Pre DOAC TTR**				
<65%	3699	58.4	276	2.1
**_e_** **GFR**				
<15			16	5.8
≥15			9 ^b^	3.3
Missing			251	90.9
≥65%	680	10.7	1342	10.2
Missing	1439	22.7	4807	36.4
**Cardiovascular event**				
**Post VKA TEE**	2589	40.9	288	2.2
**_e_** **GFR**				
<15			13	4.5
≥15			9 ^b^	3.1
Missing			266	92.4
**ICH**	404	6.4	58	0.4
**_e_** **GFR**				
<15			2	3.4
≥15			2 ^b^	3.4
Missing			54	93.1
**SHRB**	1754	27.7	80	0.6
**_e_** **GFR**				
<15			17	21.3
≥15			3 ^b^	3.8
Missing			60	75.0

**DOACs**: direct acting oral anticoagulants; **VKA**: vitamin K antagonist; **_e_GFR**: glomerular filtration estimated by CKD-EPI (mL/min/1.73 m^2^); **ADR**: adverse drug reaction; **TTR**: time in therapeutic range according to the Rosendaal method; **TEE**: thromboembolic event; **ICH**: intracranial hemorrhage; **SHRB**: stroke with high risk of bleeding; ^a^ ADR to DOACs, or DOAC prescribed as first-line anticoagulant without history of post-VKA TEE, SHRB or ICH; ^b^ also have ADR to DOACs.

**Table 7:** Adequate prescribing of vitamin K antagonists (VKAs) based in the 2016 to the IPT criteria.

	Adequate Prescribing of VKAs		Inadequate Prescribing of VKAs	
	n	%	n	%
Total	26,521	64.0	14,909	36.0
**TTR^R^**				
<65%	1379	5.2	14,732	98.8
**_e_** **GFR**				
<15	196	14.2		
≥15	1 ^b^	0.1		
Missing	1182	85.7		
≥65%	25,140	94.8	177	1.2
**ICH**			170	
			7 ^a^	
Missing	2 ^c^	0.0		
**_e_** **GFR**				
<15	313	1.2		
≥15	23,035	86.9	14,909	100
Missing	3173	12.0		
**ADR**				
VKA			13	0.1
DOAC	6	0.0	10 ^a^	0.1
**ICH**	35	0.1	329	2.2
**_e_** **GFR**				
<15	8	22.9		
Missing	27	77.1		

**VKAs**: vitamin K antagonists; DOACs: direct oral anticoagulants; **TTR^R^**: time in therapeutic range according to the Rosendaal method; **_e_GFR**: glomerular filtration estimated according to CKD-EPI (mL/min/1.73 m^2^); **ICH**: intracranial hemorrhage; **ADR**: adverse drug reaction; ^a^ ADR VKA. ^b^ **ADR** DOAC. ^c^ Insufficient INR to calculate TTR^R^.

## Data Availability

The data that support the findings of this study were obtained from SIDIAP database (Information System for Research in Primary Care). This database is representative of the Catalan population. Restrictions apply to the availability of these data, which were used under license for this study. The authors have no authorization to share the data.

## Supplementary Materials

The following are available online at https://www.mdpi.com/1660-4601/18/5/2244/s1, Figure S1: Detail of inadequate DOAC prescription.
