# Supplementary material for: Oral Anticoagulant Adequacy in Non-Valvular Atrial Fibrillation in Primary Care: A Cross-Sectional Study Using Real-World Data (Fantas-TIC Study)
Source: Int J Environ Res Public Health. 2021 Feb 24;18(5):2244. doi: 10.3390/ijerph18052244 (PMC7956646; doi:10.3390/ijerph18052244)
Supplement: Supplementary file 1 [file ijerph-18-02244-s001.pdf]

International Journal of
Environmental Research and Public Health

MDPI

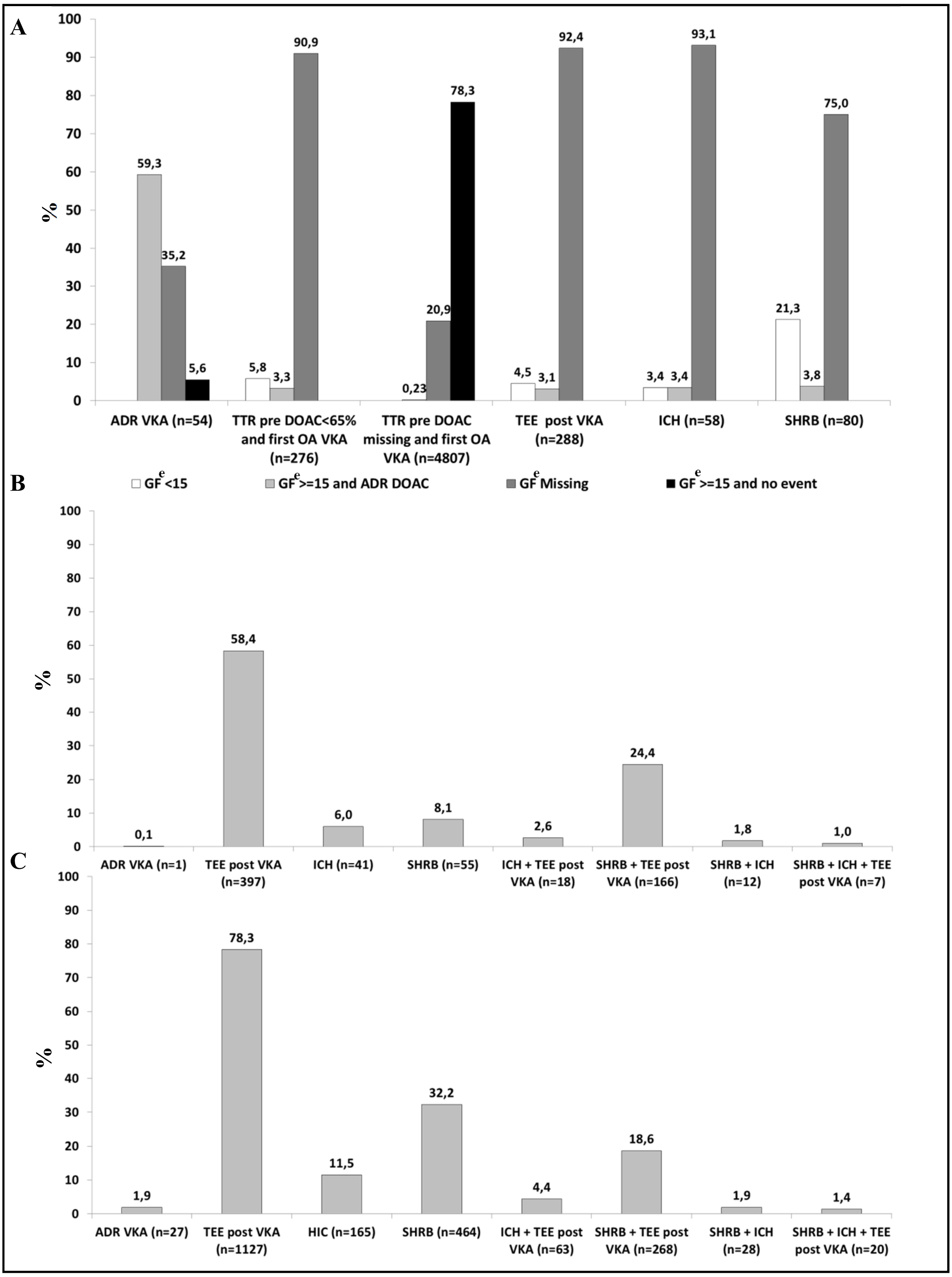

Supplementary Figure 1. (A) Detail of inadequate DOAC prescription. (B) Detail of adequate DOAC prescription, with TTR pre-DOAC and first OA VKA >=65%, cases are not excluding (n=680). (C) Detail of adequate DOAC prescription when missing value for TTR pre-DOAC and first OA VKA, cases are not excluding (n=1439). DOAC: direct oral anticoagulant. VKA: vitamin K antagonist. GF^e: glomerular filtration according to CKD-EPI (mL/min/1.73m2). ADR: adverse drug reaction; OA: Oral anticoagulants; TTR^R: time in therapeutic range according to Rosendaal method; TEE: thromboembolic event; ICH: intracranial haemorrhage; SHRB: Stroke with high risk of bleeding; No event: no TEE post VKA, ICH, SHRB.
